# Knowledge, Attitude, and Practice of Paracetamol and Ibuprofen Administration Among Caregivers of the Pediatric Age Group in Jeddah

**DOI:** 10.7759/cureus.12460

**Published:** 2021-01-03

**Authors:** Fatemah Kamel, Rania Magadmi, Noran M AbuOuf, Faisal S Alqahtani, Abdullah A Bamousa, Abdulrahman T Alqutub, Abdulrahman A Bagber, Albraa H Abdulgafor, Fayez Alzahrani, Hassan Alsafi

**Affiliations:** 1 Pharmacology, King Abdulaziz University, Jeddah, SAU; 2 Pediatrics, King Abdulaziz University, Jeddah, SAU; 3 Medicine, King Abdulaziz University, Jeddah, SAU; 4 Medicine and Surgery, King Abdulaziz University, Jeddah, SAU

**Keywords:** pain, fever management, pediatric pharmacology

## Abstract

Background

Fever is one of the most common pediatric conditions usually managed by parents and the cause of nearly all pediatrician visits. However, many parents find the management of childhood fever and febrile diseases challenging owing to a lack of understanding of the nature, effects, and therapies of fever management.

Objectives

This study aimed to assess the knowledge, attitude, and practice of paracetamol and ibuprofen administration among caregivers of the pediatric age group.

Design

Observational cross-sectional survey.

Setting

Jeddah, Saudi Arabia.

Materials and Methods

Data were collected between April 2018 and April 2019 using a pretested interviewer-administered questionnaire consisting of 40 questions.

Sample Size

Overall, 493 caregivers were interviewed.

Results

Paracetamol was reported as the most common antipyretic used by the caregivers (54%) to control fever. Ibuprofen was the least preferred drug (18.5%). The majority of the participants (51.7%) admitted administering antipyretics at a body temperature of 38-38.5°C. A total of 90.7% of the participants measured children’s temperature using a thermometer before administering antipyretics. Dosage was determined according to each child’s age (40.4%), weight (32%), or illness severity (27.6%). However, 36.7% and 51.5% of the participants were unsure of the correct dosage of paracetamol and ibuprofen, respectively. Regarding the maximum frequency of paracetamol use, only 3.7% of the participants answered correctly. Most parents (70.4%) believed that a paracetamol/ibuprofen prescription was not necessary. Overall, 97% of the sample demonstrated inadequate knowledge about antipyretic administration.

Conclusions

Most caregivers had inadequate knowledge regarding factors that influence paracetamol and ibuprofen dosage and frequency of administration. This low level of knowledge increases the risk of improper drug intake, which can result in serious side effects, thereby indicating the need for the development of educational route programs to provide parents with appropriate education and information on fever and fever management.

## Introduction

Fever is one of the most common pediatric conditions managed by parents and the cause of nearly 70% of all pediatrician visits [1]. However, although fever is a common occurrence, the management of childhood fever remains challenging for many parents [2,3]. This is because fever is not well understood by the general population; consequently, many parents find fever management overwhelming and intimidating, resulting in anxiety and concern [4-6]. According to various studies in published literature, parents have excessive conflicting information as well as misconceptions on fever management [1-4]. Moreover, many parents perceive pediatric fever as a disease rather than a sign or symptom of illness and therefore have several misconceptions about its management. Moreover, insufficient knowledge of the fever’s cause, coupled with misconceptions about its effects on a child’s health, results in excessive parental fear and anxiety regarding this condition [1].

Paracetamol, also known as acetaminophen, is an antipyretic commonly used for the management of fever and moderate to mild pain in all age groups, and it is well documented to be safe when used at the recommended doses; in many countries, it is often sold as an over-the-counter painkiller. The effectiveness of paracetamol is identical to that of aspirin; however, unlike aspirin, its anti-inflammatory activity is demonstrable. Paracetamol is also preferred over aspirin because it is less likely to irritate the stomach [7]. Furthermore, after its discovery, paracetamol use became more prevalent as it was considered a viable replacement for aspirin, especially in pediatric patients with a high risk of developing Reye syndrome as a side effect of aspirin use [8]. This makes paracetamol the first-choice medicine to treat and reduce fever in children, with most parents having used the drug since their child’s first febrile illness. Paracetamol is also the most commonly used drug for self-medication by mothers of children ≤12 years of age [7].

Although paracetamol has been widely used for fever and pain management for more than 60-70 years, over-toxicity cases have been reported [7]. It has been found that paracetamol is one of the over-the-counter medications commonly associated with unintentional overdose in children under the age of five years [9]. Paracetamol overdose has also been associated with life-threatening hepatic damage due to the excessive production of the reactive and toxic metabolite N-acetyl-p-benzoquinone imine during drug metabolism [10]. Hepatic damage is dangerous because it often passes unnoticed, especially during the first four to six days of occurrence [7]. Most severe pediatric hepatotoxicity cases were attributed primarily to cumulative toxicity resulting from repetitive doses rather than acute intoxication caused by a single massive overdose [11]. This implies that administering paracetamol at the correct dosage and time interval can substantially reduce the potential effect for hepatic damage.

A study has shown that paracetamol poisoning is one of the most common causes of acute liver failure in the United States and the United Kingdom [12]. In countries where paracetamol is widely used, several cases of acute damage to liver cells due to repeated doses of paracetamol have been reported, especially if the maximum therapeutic dose of 90 mg/kg/24 h is exceeded [12].

Ibuprofen is a non-steroidal anti-inflammatory drug [13] that reduces the activity of hormones associated with pain and inflammation in the body. It is commonly used to reduce fever and treat inflammation or pain caused by different conditions, including toothache, headache, arthritis, back pain, or minor injury. Ibuprofen overdoses lead to effects similar to those caused by paracetamol since both are painkillers [14]. Further, ibuprofen overdose has been associated with gastrointestinal tract (GIT) complications, acute renal failure, and metabolic acidosis. The severity of these side effects indicates that caregivers need to be knowledgeable about administering these antipyretics to children. Thus, here, we aimed to assess the knowledge, awareness, and practice of ibuprofen and paracetamol administration by caregivers and draw conclusions on how to implement a public awareness program for antipyretic use in pediatric patients.

## Materials and methods

Ethics approval

The study was approved by the Unit of Biomedical Ethics Research Committee at the Faculty of Medicine, King Abdulaziz University (Reference No. 75-20). Informed consent was obtained from all participants before the commencement of the interviews.

Study setting

This observational cross-sectional study included caregivers of children living in the city of Jeddah, in the western region of Saudi Arabia. Data were collected between April 2018 and April 2019.

Study population

The study sample (n=493) was selected randomly. The sampling process took place at the Red Sea Mall, the Al Salaam Mall, and King Abdulaziz University Hospital.

The inclusion criteria were as follows: caregivers of children aged 0-10 years, having managed at least one pediatric fever episode with paracetamol or ibuprofen, including parents, baby sitters, and grandparents aged 18-65 years; English or Arabic speakers; Saudi and non-Saudi caregivers; and willing to participate in the study.

Data collection

Data collection was performed by seven 6th-year medical students, who were trained how to use the survey instrument before conducting the interviews with the participants. The study’s aims and objectives were explained to the participants before enrollment in the study.

Survey instrument

An interview questionnaire that comprised 40 questions divided into four sections was used in the study. The first section inquired about the demographic data of the caregiver and the child. The second section consisted of seven questions to evaluate the caregiver’s knowledge on the use of paracetamol and ibuprofen. The third part explored the caregiver’s attitude regarding paracetamol and ibuprofen use. Finally, the fourth section explored the caregiver’s practices regarding paracetamol and ibuprofen use. English and Arabic versions of the questionnaire were prepared and reviewed by a bilingual expert. A pilot survey was also conducted on 25 randomly selected individuals to ensure the questionnaire’s reliability and internal validity. Additionally, the questionnaire was reviewed by two pharmacologists to assess its reliability.

Data analysis

Data cleaning and data analysis were performed using Statistical Package for Social Sciences (SPSS) version 21.0 (IBM Corp., Armonk, NY, USA). Frequency and percentages were used for the descriptive analysis. The cutoff point of the knowledge score was set at 5/7. Participants with scores below 5 were considered to have poor knowledge, whereas those with scores equal to or above 5 were considered to have good knowledge of the topic. Pearson’s chi-squared test was used to analyze the strength of the association between demographic data and the caregivers’ knowledge level. P < .05 was considered statistically significant.

## Results

Demographic characteristics of the study population

The total number of caregivers involved in the study was 493. As summarized in Table 1, 26.2%, 42.6%, 29.2%, and 2% of the participants aged 18-29, 30-39, 40-49, and >60 years old, respectively, were included. Most participants were female (75.5%) and citizens of Saudi Arabia (80.5%). Regarding education, most participants had a bachelor’s degree (75.5%). Regarding family status, 30.8% had three or more children, whereas the remaining participants had either one or two children. The participant employment status was as follows: 47.7% were full-time employees, 40.4% were unemployed, and 12% had a part-time job. 

**Table 1:** Caregivers’ characteristics (n=493)

		Frequency	Percentage (%)
Age	18–29	129	26.2
	30–39	210	42.6
	40–59	144	29.2
	>60	10	2
Sex	Male	121	24.5
	Female	372	75.5
Nationality	Saudi	397	80.5
	Non-Saudi	96	19.5
Education level	Elementary	4	0.8
	Intermediate	17	3.4
	Secondary	95	19.3
	University	377	76.5
Employment status	Not working	199	40.4
	Part time	59	12
	Full time	235	47.7
Number of children	1	120	24.3
	2	126	25.6
	3	95	19.3
	>3	152	30.8

Caregivers’ knowledge of antipyretic administration

Table 2 shows the items used to measure the knowledge score of the caregivers on the use of paracetamol and ibuprofen as antipyretic drugs; 40.4% of the caregivers indicated that the age of the child was the main factor influencing the dosage of paracetamol and ibuprofen administered, whereas 32% reported the weight of the child as the main factor.

**Table 2:** Caregivers’ knowledge of antipyretic administration (n=493)

Questions		Frequency	Percentage (%)
Do you know the factors influencing the paracetamol/ ibuprofen dose?	Child's weight (correct answer)	158	32
	Child's age	199	40.4
	Illness severity (body temperature in °C)	136	27.6
What is the recommended dose for paracetamol?	<10–15 mg/kg/dose	139	28.2
	10–15 mg/kg/dose (correct answer)	159	32.3
	>10–15 mg/kg/dose	14	2.8
	Not sure	181	36.7
How often do you administer paracetamol?	Every 4 hours	66	13.4
	Every 6 hours (correct answer)	149	30.2
	Every 8 hours	160	32.5
	Whenever the body temperature exceeds 37°C	118	23.9
What is the maximum daily frequency of paracetamol administration (times)?	1	21	4.3
	2	58	11.8
	3	187	37.9
	4	164	33.3
	5 (correct answer)	18	3.7
	6	45	9.1
What is the recommended dose for ibuprofen?	<4–10 mg/kg/dose	49	9.9
	4–10 mg/kg/dose (correct answer)	155	31.4
	>4–10 mg/kg/dose	35	7.1
	Not sure	254	51.5
How often do you administer ibuprofen?	Every 4 hours	46	9.3
	Every 6 hours	107	21.7
	Every 8 hours (correct answer)	199	40.4
	Whenever the body temperature exceeds 37°C	141	28.6
What is the maximum daily frequency of ibuprofen administration (times)?	1	26	5.3
	2	126	25.6
	3 (correct answer)	195	39.6
	4	105	21.3
	5	11	2.2
	6	30	6.1

Of all participants, 32.3% were aware of the recommended dosage of paracetamol (10 to 15 mg/kg), whereas 36.7% were not sure. Of the total number of caregivers, 30.2% administered paracetamol every six hours compared to 32.5% who administered it every eight hours. A staggering 23.9% administered paracetamol to their child whenever the child’s body temperature exceeded 37°C. As for the frequency of the daily intake of paracetamol, only 3.7% specified a daily maximum of five times, compared to 37.9% reporting a daily maximum of three times and 33.3% of four times.

Of all caregivers using ibuprofen, 31.4% reported administering 4-10 mg/kg of ibuprofen as recommended, whereas 51.5% were not sure about the dosage. Regarding the time interval of administration, 40.4% administered ibuprofen every eight hours, 21.7% every six hours, and 9.3% every four hours; 28.6% administered it whenever the child’s body temperature exceeded 37°C. As for the frequency of ibuprofen intake, 39.6% specified a daily maximum of three times, compared to 25.6% reporting a maximum of two times and 21.3% of four times.

Only 32% of the participants answered correctly when questioned about the factors that influence paracetamol and ibuprofen dosage (Table 3). The results about the dosage and frequency pertaining to paracetamol and ibuprofen usage were as follows: 30.2% answered correctly regarding the frequency of administering paracetamol, whereas 40.4% answered correctly regarding the frequency of administering ibuprofen. Only 3.7% answered correctly regarding the maximum daily frequency of paracetamol compared to the 39.6% who answered correctly regarding ibuprofen frequency.

**Table 3:** Caregivers’ knowledge score (n = 493)

Knowledge items	False		True	
	Frequency	Percentage (%)	Frequency	Percentage (%)
Factors influencing paracetamol/ibuprofen dose	335	68	158	32
Recommended dose for paracetamol	334	67.7	159	32.3
How often is paracetamol administered?	344	69.8	149	30.2
Maximum daily frequency of paracetamol	475	96.3	18	3.7
Recommended dose for ibuprofen	338	68.6	155	31.4
How often is ibuprofen administered?	294	59.6	199	40.4
Maximum daily frequency of ibuprofen	298	60.4	195	39.6
Overall knowledge score (Mean [SD], 2.1 [1.34])				
Poor knowledge (<5 score)	478 (97%)			
Good knowledge (≥5 score)	15 (3.0%)			

Therefore, the mean for the overall knowledge score was 2.09, with only 3% of the participants presenting decent knowledge and 97% demonstrating poor knowledge of the factors influencing paracetamol and ibuprofen dosage.

Caregivers’ awareness of antipyretic administration

The caregivers’ awareness level is presented in Table 4. Approximately 71.2% of the caregivers stated that they obtained information on paracetamol and ibuprofen from doctors and 20.3% from pharmacists. Of all caregivers, 25.8% stated that they were aware of liver damage as a side effect of paracetamol. Regarding the route/form used to administer paracetamol and ibuprofen, 52.1% of caregivers used syrups, whereas 38.1% of caregivers used a combination of both syrups and suppositories.

**Table 4:** Caregivers’ awareness on antipyretic administration (n=493)

		Frequency	Percentage (%)
From where do you get information regarding paracetamol and ibuprofen?	Doctor	351	71.2
	Pharmacist	100	20.3
	Media	12	2.4
	Other parents	22	4.5
	Other	8	1.6
Are you aware of paracetamol’s side effects?	Liver damage	127	25.8
	Kidney damage	99	20.1
	Allergy	50	10.1
	Effect on stomach	24	4.9
	Other	193	39.1
What is the form of paracetamol/ibuprofen that you use?	Syrups	257	52.1
	Suppositories	41	8.3
	Injection	7	1.4
	Syrups and suppositories combined	188	38.1
Do you think it is fine to give paracetamol/ibuprofen to your child without a prescription?	No	146	29.6
	Yes	347	70.4
Do you think it is fine to give paracetamol/ibuprofen together?	Yes	74	15
	No	419	85

Regarding the caregivers’ awareness about administering paracetamol/ibuprofen without a prescription, 70.4% stated that a prescription was not needed and only 29.6% stated that it was warranted. Approximately 85% of the caregivers thought that paracetamol and ibuprofen should not be administered together, whereas only 15% thought that they can be given together.

Caregivers’ practices regarding antipyretic administration

Of the caregivers, 51.7% claimed that they administered antipyretics at a body temperature of 38-38.5°C, whereas 32.9% reported that they administered antipyretics when the temperature exceeded 38.5°C. Only 14.2% and 1.2% administered antipyretics for temperatures greater than 39°C and 40°C, respectively. Regarding the instruments or devices used to measure body temperature, 74.6% used a thermometer, 23.1% judged by touching the forehead, whereas less than 2.2% judged by touching the lips or used other techniques to estimate the temperature.

Regarding the caregivers’ practices (Table 5), most participants agreed on paracetamol being the most common antipyretic administered to children (54%), 18.5% reported ibuprofen as the most common antipyretic, and 14.8% stated that both were equally common; only 12.8% thought that there were other more common antipyretics. A total of 35.9% of caregivers administered paracetamol/ibuprofen for reasons other than fever and 90.7% measured the child’s body temperature using a thermometer before administering antipyretics. The majority (85.2%) read the medication leaflet beforehand, whereas 14.8% did not. Measuring spoons and syringes were the most commonly used measuring devices when administering paracetamol/ibuprofen with frequencies of 42.8% and 43.8%, respectively.

**Table 5:** Caregiver practices in antipyretic administration (n=493)

		Frequency	Percentage (%)
At which body temperature do you administer an antipyretic?	38–38.5	255	51.7
	>38.5	162	32.9
	>39	70	14.2
	>40	6	1.2
How do you measure the child’s body temperature?	Using a thermometer	368	74.6
	By touching the forehead	114	23.1
	By touching the lips	8	1.6
	Other	3	0.6
The most common antipyretic that you administer to your child	Paracetamol	266	54
	Ibuprofen	91	18.5
	Both	73	14.8
	Other	63	12.8
Do you administer paracetamol/ibuprofen for reasons other than fever?	Yes	177	35.9
	No	316	64.1
Do you measure the child’s body temperature using a thermometer before administering antipyretics?	Yes	447	90.7
	No	46	9.3
Do you read the medication leaflet?	Yes	420	85.2
	No	73	14.8
What do you use as a measuring device when you administer paracetamol/ibuprofen?	Teaspoon	41	8.3
	Measuring spoon	211	42.8
	Measuring syringe	216	43.8
	Other	25	5.1

Factors affecting caregivers’ level of knowledge

Caregivers aged 30-39 years had a good level of knowledge compared to those >60 years old (Table 6). However, the difference was not significant (P=.803).

**Table 6:** Demographic factors affecting caregivers’ knowledge (n=493)

Demographic characteristics		Knowledge level		P value
		Good N (%)	Poor N (%)	
Age	18–29	3 (2.3)	126 (97.7)	.803
	30–39	8 (3.8)	202 (96.2)	
	40–59	4 (2.8)	140 (97.2)	
	>60	0 (0)	10 (100)	
Sex	Male	3 (2.5)	118 (97.5)	.409
	Female	12 (3.2)	360 (96.8 )	
Nationality	Saudi	13 (3.3)	384 (96.7)	.746
	Non-Saudi	2 (2.1)	94 (97.9)	
Education level	Elementary	0 (0)	4 (100)	.03*
	Intermediate	0 (0)	17 (100)	
	Secondary	0 (0)	95 (100)	
	University	15 (4)	369 (96)	
Employment status	Not working	4 (2)	195 (98)	.17
	Part time	1 (1.7)	58 (98.3)	
	Full time	10 (4.3)	225 (95.7)	
Number of children	1	1 (0.8)	119 (99.2)	.22
	2	5 (4)	121 (96)	
	3	7 (7.4)	88 (92.6)	
	>3	2 (1.3)	150 (98.7)	

Similarly, sex and nationality did not affect the level of knowledge (P=.409 and P=.746, respectively). However, female Saudi caregivers showed a slightly higher knowledge level compared with peer controls.

Conversely, caregivers with a university education level showed a significantly better knowledge level regarding antipyretics than the other groups (P=.03).

Finally, the employment status and number of offspring were not found to be statistically significant factors affecting the knowledge level (P=.17, P=.22, respectively). However, full-time caregivers and those with three children showed a slightly better level of knowledge.

## Discussion

This study evaluated caregivers' knowledge, awareness, and practice of the dosage, side effects, and complications of over-the-counter antipyretic drug administration to the pediatric age group in Jeddah, Saudi Arabia.

Regarding fever measurement, our results indicate that a large number of parents use a thermometer as the primary method, whereas other techniques such as touching the forehead or the lips were less commonly used. These findings are similar to those from other studies [1,15]. Surprisingly, most parents in these studies measured fever by touching the forehead, partly due to the poor socio-economic condition in those countries, [7,15], which poses significant difficulties for parents in purchasing thermometers.

Herein, we found that paracetamol was the most common antipyretic that parents used to treat fever which was corroborated by research conducted in other countries [1,2,16,17]. Moreover, since many parents pass on knowledge/inform their child’s caregiver about paracetamol use for fever, we believe that they do not provide adequate information and correct knowledge about dosage or how/when to administer it, explaining why 97% of caregivers in our study presented an inadequate knowledge score. Only 32.2% of caregivers were only aware of the correct dose of 10 mg/kg/day when questioned about paracetamol dosage. Conversely, 58.3% responded correctly on this topic in another study [12]. This variation may be attributed to a lack of information on the daily prescription dose of paracetamol. These findings align with those from other studies conducted in Sri Lanka, Nigeria, and Australia [7-9], which highlighted the need for parental education regarding fever medication dosage and administration.

This low level of knowledge about the dosage and administration of antipyretics may influence improper drug intake, which can cause serious side effects. Furthermore, children of working mothers being taken care of by babysitters are at a higher risk of antipyretic overdosing, as found in another study [7].

Here, we also found that most parents received information from doctors and pharmacists and read the medication leaflet beforehand. However, these affirmations were not consistent with the knowledge scores since most participants achieved a low score in this domain. Similar paradoxical results have been found in another study [7]. This may indicate that although parents may read the drug information leaflet before administering medications to their children, they may have a limited understanding of the information read. Hence, this may result in a positive cautious behavior of reading the leaflet, which unfortunately does not necessarily mean correct administration, due to a lack of understanding. Consequently, this aspect is worthy of further investigation.

Walsh et al. [9] mentioned that health professionals play an important role in providing parents with appropriate strategies regarding fever management, which in turn requires the provision of evidence-based education to health professionals. However, our study found that most parents (70.4%) administered antipyretics without a prescription. Conversely, 46.8% of parents in another study claimed to administer antipyretics based on a doctor’s prescription [15]. This disparity could be explained as parents in our population underestimating the damaging effects of paracetamol and ibuprofen. Therefore, they tend to administer paracetamol based on their experience. 

The caregivers’ educational status had a significant effect on the knowledge and forms used when administering antipyretics, a finding that corroborates with those of other studies including different populations [17-20]. Concomitantly, these results may indicate that caregivers with higher education may better comprehend the information on a drug leaflet, thus improving their knowledge of administration. Future research is warranted to elucidate these effects.

Regarding caregivers’ awareness of the side effects associated with antipyretics, 56% of the participants specified certain side effects of the drugs, whereas the rest believed that they caused none or unknown effects; 25.8%, 20.1%, and 10.1% of caregivers believed that the drugs caused liver toxicity, kidney damage, and allergies, respectively. We believe that this is a small number of the population who were conscious of the side effects of both drugs, which may warrant future studies and awareness campaigns. Further, syrups were the most preferred form used to administer antipyretics (52.1%). This finding is similar to that of another survey [15], which showed that parents favored oral drugs (51.2%) over suppositories. This might be because the oral route is convenient for the administration of medication in children.

We found that the number of children the parents/caregivers did not influence their knowledge score. These results are in contrast with those from a study conducted in Denmark [2] showing that parents who have more than one child were better prepared, had less fear, and greater knowledge when using antipyretics as a result of previous experience.

Finally, this survey assessed parents’ responses related to the body temperature at which they administered antipyretics. More than 50% claimed they would start at the lowest margin, which was 38°C. This finding is similar to that from other studies [1,9] in which parents defined fever as any temperature above 38°C and would, therefore, start medication at this level.

Strengths and limitations

One of the main strengths of the study is the large sample size, which strongly contributes to the generalizability of the results. The manual data input used for each parent to prevent misunderstanding of the survey questions is another strength, contributing to the results’ reliability. Another strength is the random selection of participants at malls and clinics. Moreover, we ensured that the survey was available in both Arabic and English to ensure that both Saudi and non-Saudi caregivers could be interviewed, contributing to the variability of participants included. However, one of the limitations of the study is that the collected data concerned residents of the same city, Jeddah; results from other territories may show different knowledge, practice, and attitude scores. Hence, the results may not be representative of the entire Saudi Arabian population. As such, this indicated the necessity for further surveys in other cities. Additionally, since this study was developed based on previous research on the same subject, it was discovered that there is a limited number of studies in Jeddah and worldwide, on the side effects of antipyretics as well as the awareness of correct dosages and general knowledge about these drugs. 

## Conclusions

This study highlights the need for parental education on the use, dosage, and side effects of antipyretic drugs. Hence, this work demonstrated that most parents were unaware of the severity of the side effects of paracetamol and ibuprofen. Furthermore, more than half of the parents did not know the correct dosage of both drugs, indicating a lack of knowledge regarding the use of antipyretics for fever management. It also showed that the caregivers who knew the actual side effects and dosage had a bachelor's degree, which shows that educational background plays a significant role in parental knowledge of fever management. The majority of parents used syrups as the form of administering antipyretics. There were no significant differences in knowledge scores between Saudis and non-Saudis. Additionally, the researchers felt that parents often panic when treating their child due to the fear of the harmful effect of fever, which may lead to unintentional overdoses. It found that the number of offspring that parents have do not influence parental knowledge about antipyretics. Our findings thus indicate a need for educational programs to provide parents with proper education on fever and fever management.

## Appendices

**Table 7:** Part 1. Demographic Data of caregiver

1	Age of the caregiver:	18-29	30-39	40-59	³60	
2	Gender of the caregiver:	Male	Female			
3	Nationality:	Saudi	Non-Saudi			
4	Education level of the caregiver:	Non educated	Elementary	Intermediate	Secondary	University
5	Employment status:	Not working	Part time	Full time		
6	Number of children	1	2	3	>3	
7	Child’s age (the sick one):	0-3 months	4-36 months	>36 months		

**Table 8:** Part II: Knowledge of caregivers regarding paracetamol and ibuprofen

8	At which temperature you give antipyretic?	38-38.5	>38.5	>39	>40	
9	How do you measure child’s temperature?	By thermometer	By touching the forehead	By touching the lip	other	
10	From where do you get the information regarding paracetamol and ibuprofen dose?	Doctor	Pharmacist	Media	Other parents	Others
11	Are you aware of paracetamol side effects?	Liver damage	Kidney damage	Allergy	Effect on stomach	Other
12	Do you know the factors influencing paracetamol/ ibuprofen dose?	Child’s weight	Child’s age	Illness severity (degree of temp)		

**Table 9:** Part III: Attitude of caregivers regarding paracetamol and ibuprofen

13	What is the form of paracetamol/ ibuprofen you use?	Syrups	Suppositories	Injection	Syrups and suppositories combined
14	Do you think it is ok to give paracetamol/ ibuprofen to your child without prescription?	Yes	No		
15	Do you think it is ok to give paracetamol/ ibuprofen together?	Yes	No		

**Table 10:** Part IV: Practice of caregivers regarding paracetamol and ibuprofen

16	Most common antipyretic you give your child?	Paracetamol	Ibuprofen	Both	Other	
17	What is the recommended dose for paracetamol?	<10-15 mg/kg/dose	10-15 mg/kg/dose	<10-15 mg/kg/dose	Not sure	
18	How often do you give paracetamol?	Every 4 hr	Every 6 hr	Every 8 hr	Whenever temperature exceed 37	
19	What is the maximum daily frequency of paracetamol?	2	3	4	5	6
20	What is the recommended dose for ibuprofen?	<4-10 mg/kg/dose	4-10 mg/kg/dose	< 4-10mg/kg/dose	Not sure	
21	How often do you give ibuprofen?	Every 4 hr	Every 6 hr	Every 8 hr	Whenever temperature exceed 37	
22	What is the maximum daily frequency of ibuprofen?	2	3	4	5	6
23	Do you give paracetamol/ ibuprofen for other reasons (OTHER THAN FEVER)?	Yes	No			
24	Do you measure child’s temperature by thermometer before giving the antipyretic?	Yes	No			
25	Do you have the habit to read the medication leaflet?	Yes	No			
26	What do you use as a measuring device when you give paracetamol/ ibuprofen?	Tea spoon	Measuring spoon	Measuring syringe	Other	

**Table 11:** الجزء الأول :البيانات الديموغرافية المتعلقة بالمربين/المربيات

	³60	40-59	30-39	18-29	ـ عمر مقدم الرعاية :	
			أنثى	ذكر	ـ جنس مقدم الرعاية :	
			غير سعودي	سعودي	ـ الجنسية :	
جامعي	ثانوي	متوسط	ابتدائي	غير متعلم	ـ المستوى التعليمي لمقدم الرعاية :	
		دوام كلي	دوام جزئي	عاطل	- الحالة الوظيفية :	
	>3	3	2	1	-عدد الأطفال :	
		>36 شهر	4-36 أشهر	0-3 أشهر	- عمر الطفل ( المريض) :	

**Table 12:** الجزء الثاني:مدى معرفة مقدمي الرعاية عن أدوية الباراسيتامول (بانادول)وأيبوبروفين:

8	-عند أي درجة حرارة يتم إعطاء مخفض الحرارة؟	∕38-38.5	∕أكثر من38,5	∕أكثر من39	∕أكثر من40	
9	- كيف تقيس/ين درجة حرارة الطفل؟	∕عن طريق الثيرمومتر(جهاز لقياس الحرارة)	∕عن طريق لمس الجبين	∕عن طريق لمس الشفاه	∕أخرى	
10	-من أين تحصل/ين على المعلومات المتعلقة بجرعة (الباراسيتامول والأيبوبروفين)؟	∕ الطبيب	∕الصيدلي	∕وسائل التواصل الاجتماعي/الإعلام	∕أفراد من العائلة	∕آخرون
11	-هل أنت مدرك/ة بالأعراض الجانبية للباراسيتامول؟	∕مضرة الكبد	∕مضرة الكلية	∕مضرة المعدة	∕الحساسية	∕أخرى
12	-هل أنت مدرك/ة بالعوامل المؤثرة في اختيار جرعات (الأيبوبروفين والباراسيتامول)؟	∕وزن الطفل	∕عمر الطفل	∕شدة المرض(بالاعتماد على درجة الحرارة(		

**Table 13:** الجزء الثالث:توجه مقدمي الرعاية فيما يتعلق بالباراسيتامول والأيبوبروفين:

∕شراب وتحاميل معا	∕عن طريق الحقن	∕تحاميل	∕شراب	- ما هي الشكل الدوائي (الباراسيتامول/الأيبوبروفين) التي تستخدمها؟	13
		∕لا	∕نعم	-هل تعتقد/ين أنه من الصحيح إعطاء الطفل (أيبوبروفين/باراسيتامول) بدون إستشارة الطبيب؟	14
		∕لا	∕نعم	-هل تعتقد/ين أنه من الصحيح إعطاء (الأيبوبروفين والباراسيتامول معا)؟	15

**Table 14:** الجزء الرابع : ممارسات مقدمي الرعاية المتعلقة باعطاء (الباراسيتامول والآيبوبروفين):

	∕اخرى	∕الاثنين	∕ايبوبروفين	∕باراسيتامول	-ما هو أكثر خافض حرارة تستخدمه لطفلك؟	16
	∕غير متأكد	∕أكثر من ١٠-١٥ ميليغرام	∕١٠-١٥ ميليغرام	∕أقل من ١٠-١٥ ميليغرام	-ما هي الجرعة الموصى بها للباراسيتامول؟	17
	∕عندما تزيد درجة الحرارة عن ٣٧	∕ كل ٨ ساعات	∕كل ٦ ساعات	∕ كل ٤ ساعات	-كم عدد المرات التي يتم فيها إعطاء الباراسيتامول؟	18
6∕	5∕	4∕	3∕	1∕	-ما هو الحد الأقصى اليومي لتكرار اعطاء الباراسيتامول؟	19
	∕غير متأكد	∕أكثر من ٤-١٠ ميليغرام	∕٤-١٠ ميليغرام	∕ أقل من٤-١٠ ميليغرام	-ما هي الجرعة الموصى بها للأيبوبروفين؟	20
	∕ لمن تزيد درجة الحرارة عن ٣٧	∕كل ٨ ساعات	∕كل ٦ ساعات	∕ كل ٤ ساعات	--كم عدد المرات التي يتم فيها إعطاء الأيبوبروفين؟	21
6∕	5∕	4∕	3∕	2∕	-ما هو الحد الأقصى اليومي لتكرار اعطاء الأيبوبروفين؟	22
			∕لا	∕نعم	-هل تقوم بإعطاء الباراسيتامول-أيبوبروفين لأسباب أخرى(لغير الحمى)؟	23
			∕لا	∕نعم	-هل تقوم بقياس درجة حرارة الطفل قبل اعطائه خافض الحرارة؟	24
			∕لا	∕نعم	-هل تحرص عادة بقراءة المنشور المرفق بالدواء؟	25
	∕ اخرى	∕ابرة قياسية	∕ملعقة قياسية	∕ملعقة شاي	-ما هي الادوات التي تستخدمها لقياس جرعة الباراسيتامول –أيبوبروفين عندإعطائها للطفل؟	26
